# Impact of goal-directed hemodynamic management on the incidence of acute kidney injury in patients undergoing partial nephrectomy: a pilot randomized controlled trial

**DOI:** 10.1186/s12871-021-01288-8

**Published:** 2021-03-03

**Authors:** Qiong-Fang Wu, Hao Kong, Zhen-Zhen Xu, Huai-Jin Li, Dong-Liang Mu, Dong-Xin Wang

**Affiliations:** 1 Department of Anesthesiology and Critical Care Medicine, Peking University First Hospital, Beijing, 100034 China; 2 Outcomes Research Consortium, Cleveland, OH USA

**Keywords:** Partial nephrectomy, Hemodynamic management, Acute kidney injury

## Abstract

**Background:**

The incidence of acute kidney injury (AKI) remains high after partial nephrectomy. Ischemia-reperfusion injury produced by renal hilum clamping during surgery might have contributed to the development of AKI. In this study we tested the hypothesis that goal-directed fluid and blood pressure management may reduce AKI in patients following partial nephrectomy.

**Methods:**

This was a pilot randomized controlled trial. Adult patients who were scheduled to undergo partial nephrectomy were randomized into two groups. In the intervention group, goal-directed hemodynamic management was performed from renal hilum clamping until end of surgery; the target was to maintain stroke volume variation < 6%, cardiac index 3.0–4.0 L/min/m^2^ and mean arterial pressure > 95 mmHg with crystalloid fluids and infusion of dobutamine and/or norepinephrine. In the control group, hemodynamic management was performed according to routine practice. The primary outcome was the incidence of AKI within the first 3 postoperative days.

**Results:**

From June 2016 to January 2017, 144 patients were enrolled and randomized (intervention group, *n* = 72; control group, n = 72). AKI developed in 12.5% of patients in the intervention group and in 20.8% of patients in the control group; the relative reduction of AKI was 39.9% in the intervention group but the difference was not statistically significant (relative risk 0.60, 95% confidence interval [CI] 0.28–1.28; *P* = 0.180). No significant differences were found regarding AKI classification, change of estimated glomerular filtration rate over time, incidence of postoperative 30-day complications, postoperative length of hospital stay, as well as 30-day and 6-month mortality between the two groups.

**Conclusion:**

For patients undergoing partial nephrectomy, goal-directed circulatory management during surgery reduced postoperative AKI by about 40%, although not significantly so. The trial was underpowered. Large sample size randomized trials are needed to confirm our results.

**Trial registration:**

Clinicaltrials.gov identifier: NCT02803372. Date of registration: June 6, 2016.

**Supplementary Information:**

The online version contains supplementary material available at 10.1186/s12871-021-01288-8.

## Background

Partial nephrectomy through an open incision or laparoscopic way is increasingly used to treat renal tumor with benefits of sparing nephrons and preserving renal function [1]. However, removal of renal parenchyma [2, 3], suture damage and ischemia-reperfusion injury [4] during partial nephrectomy all compromise renal function. The reported incidence of acute kidney injury (AKI) after partial nephrectomy ranged from 16.5 to 42%, and even up to 54% in solitary kidney patients [5–7]. In the study of Rajan et al. [8], 39% of patients developed AKI after partial nephrectomy; specifically, 33% had stage 1, 4% had stage 2, and 2% had stage 3 AKI after surgery. The occurrence of postoperative AKI is significantly associated with increased risks of renal function decline and chronic kidney diseases [6, 9], as well as adverse cardiovascular events and even mortality [10].

Many effects have been performed to preserve residual renal function, such as improving surgical skill, sparing more normal nephrons and shortening ischemic duration. Furthermore, improving renal tissue perfusion and alleviating ischemia-reperfusion injury (IRI) caused by renal hilum clamping may also provide renal protection. Indeed, stroke volume guided fluid infusion and inotropic therapy improves global oxygen delivery, microvascular flow and tissue oxygenation [11]. However, evidence regarding the effect of circulatory management on AKI development after partial nephrectomy is limited. Available studies mainly focused on major abdominal surgeries and gave conflicting results. For example, Pearse et al. [12] found that cardiac output–guided hemodynamic management did not reduce complications; whereas Futier et al. [13] reported that individualized blood pressure management reduced postoperative organ dysfunction. Partial nephrectomy usually involves renal hilum clamping and declamping, similar to kidney transplantation to some extent. Kidney transplantation represents a typical situation of ischemia-reperfusion; it is recommended to maintain high central venous pressure (CVP > 8 mmHg) and high mean arterial pressure (MAP > 95 mmHg) with crystalloid hydration and vasoactive drugs at the time of hilum declamping in order to improve reperfusion [14–18]. As a dynamic parameter, stroke volume variation can be used to replace central venous pressure in evaluating volume status [19]. We hypothesized that a similar strategy of crystalloid hydration and hemodynamic management based on stroke volume monitoring during partial nephrectomy might also protect kidney.

The purpose of this pilot randomized trial was to test the effects of goal-directed fluid and blood pressure management on the incidence of AKI in patients following partial nephrectomy for renal cancer.

## Methods

### Study design

This pilot randomized controlled trial which was performed in a tertiary hospital in Beijing, China. The study protocol was approved by the Clinical Research Ethics Committee of Peking University First Hospital (2016[1118]) and was a priori registered with ClinicalTrials.gov (NCT02803372) on June 6, 2016. Written informed consents were obtained from all participants.

### Participants

Potential participants were screened the day before surgery. The inclusion criteria were adult (≥ 18 years) patients scheduled to undergo elective laparoscopic or open partial nephrectomy. Patients who met any of the following criteria were excluded: (1) severe renal function impairment (estimated glomerular filtration rate [eGFR] < 45 ml/min/1.73 m^2^), (2) arrhythmia or impaired cardiac function (New-York Heart Association classification ≥ III), (3) bilateral renal surgery, (4) solitary kidney, (5) anticipated massive blood loss (≥ 800 ml) and requirement of artificial colloid infusion, or (6) American Society Anesthesiologist classification ≥ IV.

Baseline data were collected after obtaining written informed consents and included demographic variables, previous comorbidities, results of important laboratory tests, results of tumor examination, American Society of Anesthesiologists classification, and Preoperative Aspects and Dimensions Used for an Anatomical (PADUA) score. PADUA score is a simple anatomical system used to predict the risk of perioperative surgical and medical complications in patients undergoing nephron-sparing surgery; the score ranges from 6 to 14, a score ≥ 8 indicates high risk of complications [20].

### Randomization and blinding

Patients were randomly allocated to either the intervention group (goal-directed hemodynamic management) or the control group (routine hemodynamic management) in a 1:1 ratio according to computer-generated random numbers. The allocation was sealed in opaque envelopes until shortly before anesthesia induction. Randomization and group assignment were performed by a study coordinator who did not participate in perioperative care and data collection. Anesthesiologists who were responsible for anesthetic management were not involved in follow-up. Investigators who performed postoperative follow-up and patients were masked from study group assignment.

### Intervention, anesthesia and perioperative care

Routine intraoperative monitoring included electrocardiogram, non-invasive blood pressure, pulse oxygen saturation, end-tidal carbon dioxide, volatile anesthetic concentration, bispectral index, and urine output. Invasive blood pressure was monitored after anesthesia induction. Intraoperative blood pressure and heart rate were recorded automatically every 10 s by the Anesthesia Information System. Patients in the intervention group were connected to a LiDCO^rapid^ monitor (LiDCO Ltd., HM81–01, UK) which continuously displayed hemodynamic variables including stroke volume variation (SVV) and cardiac index (CI).

No premedication was administered. General anesthesia was performed for all patients. Anesthesia was induced with midazolam, propofol/etomidate, sufentanil, and rocuronium; and maintained with intravenous propofol, remifentanil/sufentanil, rocuronium/cisatracurium, and 50% nitrous oxide inhalation. The target was to maintain bispectral index between 40 and 60. Patients were ventilated through an endotracheal tube or a laryngeal mask airway with a tidal volume of 8–10 ml/kg.

For patients in the intervention group, goal-directed hemodynamic management was performed from renal hilum clamping until end of surgery. The target was to maintain a SVV < 6%, a CI between 3.0 and 4.0 L/min/m^2^, and a MAP > 95 mmHg with crystalloid fluids and intravenous infusion of dobutamine and/or norepinephrine. Volume loading with 250-ml crystalloid fluid was rapidly infused to achieve SVV < 6%. Dobutamine was infused from 2 μg/kg/min and adjusted by anesthesiologists to achieve the target of CI and MAP. Ephedrine was also administered to achieve this target when necessary. In case that hemodynamic target was not achieved or heart rate > 120% of baseline or > 100 beats per minute, norepinephrine infusion was added and adjusted. For patients in the control group, hemodynamic management was performed according to routine practice, i.e., blood pressure was maintained within 20% from baseline and a urine output > 0.5 ml/kg/h with crystalloid fluids and intravenous injection of ephedrine.

Laparoscopic partial nephrectomy was performed through retropneumoperitoneum with a carbon dioxide pressure of 12–14 mmHg. Open partial nephrectomy was performed when the laparoscopic way was not applicable. Surgery was performed with patients in the lateral position or, in some cases, in the supine position. As a routine practice, renal artery clamping was applied during resection of renal parenchyma. Diuretics such as mannitol and/or furosemide were administered at the discretion of attending surgeons. Dexamethasone (5–10 mg) and tropisetron (5 mg) could be administered to prevent postoperative nausea and vomiting. Non-steroid anti-inflammatory drugs and artificial colloids were not allowed in both groups. Blood transfusion was provided when considered necessary.

After surgery, analgesia were provided with a patient-controlled analgesia pump which was established with sufentanil (1.25 μg/ml) or morphine (0.5 mg/ml) and programmed to administer 2-ml boluses with a lockout interval of 6–8 min and a background infusion rate at 1 ml/h. Patients were encouraged to eat and drink the day of surgery. Diuretics were provided when considered necessary.

### Outcome assessment

Patients were followed up daily during the first 3 postoperative days, then weekly until 30 days after surgery, and at 3 and 6 months after surgery. Telephone interview was performed for patients after hospital discharge (Supplementary File 1). The primary endpoint was the incidence of AKI within the first 3 postoperative days. The occurrence of AKI was diagnosed according to the serum creatinine level based on the Kidney Disease: Improving Global Outcomes (KDIGO) definition [21], i.e., an increase in serum creatinine by > 0.3 mg/dl (> 26.5 μmol/l) within 48 h, or an increase in serum creatinine to > 1.5 times from baseline within 3 days.

The secondary endpoints included stages of AKI, postoperative length of hospital stay, postoperative complications within 30 days, and 30-day and 6-month mortality. The stage of AKI was classified according to the KDIGO criteria: stage 1, serum creatinine 1.5–1.9 times baseline or increase by ≥0.3 mg/dl within 48 h; stage 2, serum creatinine 2–2.9 times baseline; and stage 3, serum creatinine 3 times baseline or ≥ 4.0 mg/dl (≥ 353.6 μmol/l) or initiation of renal replacement therapy [21]. An exploratory endpoint was estimated glomerular filtration rate (eGFR) on postoperative days 1, 2, and 3 which was calculated with the Chronic Kidney Disease Epidemiology Collaboration (CKD-EPI) equation [22].

### Statistical analysis

#### Sample size calculation

Previous studies showed that AKI developed in 42% of patients after partial nephrectomy [5]. In patients recovering from major abdominal surgery, the incidence of AKI was decreased by up to 60% with optimized hemodynamic strategy [11, 13]. We assumed that the incidence of AKI following partial nephrectomy would be reduced to 20% in patients with goal-directed hemodynamic management, i.e., a 52% reduction. With significance level set at 0.05 and power set at 80%, 68 patients in each group was required. We planned to enroll 72 patients per group to allow for a 5% dropout rate.

#### Outcome analysis

Baseline balance was assessed with absolute standardized difference, calculated as the absolute difference in means, medians, or proportions divided by the pooled standard deviation [23]. Baseline variables with an absolute standardized difference ≥ 0.327 (i.e., $1.96\times \sqrt{\left(\mathrm{n}1+\mathrm{n}2\right)/\left(\mathrm{n}1\times \mathrm{n}2\right)}$) were considered imbalanced and would be adjusted in all analyses when considered necessary.

The primary outcome, i.e., the incidence of AKI within 3 days after surgery, was compared with Chi-square tests, with differences between groups expressed as relative risk (95% CI). Other numeric variables were analyzed using the independent t test (data with normal distribution) or Mann-Whitney U test (data with non-normal distribution). Categorical variables were evaluated using the Chi-square test or Fisher’s exact test. Repeatedly measured variables like eGFR change over time between groups were analyzed by two-factor repeated measures ANOVA. Repeated measured hemodynamic variables (CI and SVV before and after reperfusion) within the same group were analyzed by Wilcoxon signed-rank test. A two-sided *P* <  0.05 was considered statistically significant. Analyses were performed in the intention-to-treat population. Per-protocol analysis was also performed for the primary endpoint. All analyses were performed using SPSS 25.0 software package (IBM SPSS, Chicago, IL).

## Results

**Fig. 1:**
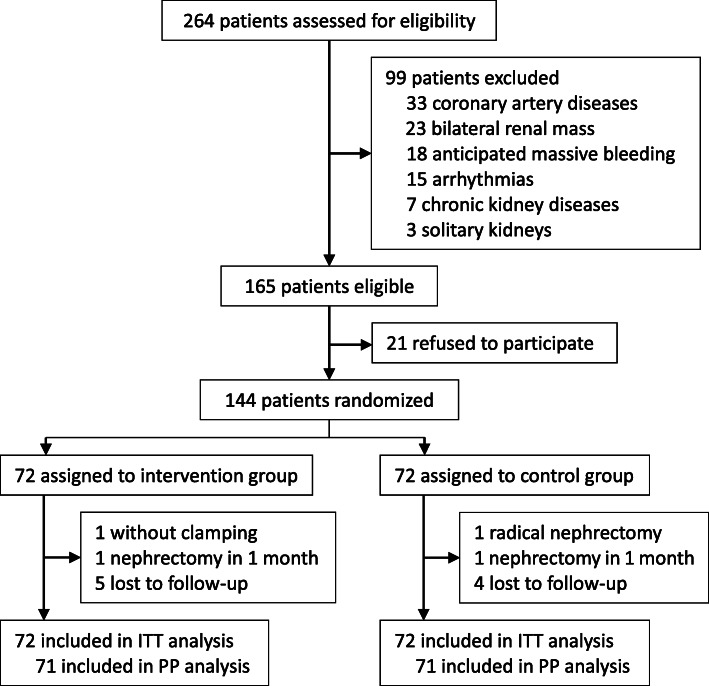
Flowchart of the study. ITT, intention-to treat. PP, per-protocol

From June 16, 2016 to January 2, 2017, 264 patients were screened for study participation. Of these, 144 patients were enrolled into the study and randomly assigned to either the intervention group (*n* = 72) or the control group (n = 72). All patients were analyzed according to the intention-to-treat principle. One patient in the intervention group did not undergo renal hilum clamping; one patient in the control group converted to radical nephrectomy. They were excluded from per-protocol analysis for the primary outcome (Fig. 1). Overall, baseline variables were well balanced between two groups except that baseline systolic blood pressure (SBP) was lower in the intervention group than in the control group (Table 1).

**Table 1:** Baseline data

	Intervention group (***n*** = 72)	Control group (***n*** = 72)	ASD
Age (years)	54.1 ± 12.1	54.5 ± 12.3	0.028
Male gender	48 (66.7%)	43 (59.7%)	0.144
Body Mass Index (kg/m^2^)	25.2 ± 3.3	25.0 ± 3.4	0.040
Comorbidity			
Hypertension	26 (36.1%)	34 (47.2%)	0.227
Diabetes	13 (18.1%)	12 (16.7%)	0.037
Respiratory diseases^a^	2 (2.8%)	7 (9.7%)	0.290
Others^b^	6 (8.3%)	5 (6.9%)	0.052
Hemoglobin (g/L)	144.1 ± 15.2	142.8 ± 14.8	0.086
Albumin (g/L)	44.9 ± 3.5	44.3 ± 4.2	0.153
eGFR (mL/min/1.73 m^2^)^c^	83 ± 18	84 ± 14	0.134
Baseline SBP (mmHg)^d^	132 ± 17	139 ± 19	**0.397**
Baseline MAP (mmHg)^d^	96 ± 13	99 ± 12	0.228
Baseline HR (bpm)^d^	73 ± 9	72 ± 10	0.084
ASA classification			0.057
1	32 (44.4%)	30 (41.7%)	
2	36 (50.0%)	38 (52.8%)	
3	4 (5.6%)	4 (5.6%)	
Maximal mass diameter (cm)	2.8 (2.2, 4.0)	3 (2.1, 4.2)	0.109
PADUA score^e^	7.5 ± 1.1	7.5 ± 1.0	0.013
Histology of cancer^f^			0.092
Clear cell carcinoma	49 (68.1%)	50 (69.4%)	
Angiomyolipoma	15 (20.8%)	9 (12.5%)	
Others^g^	8 (11.1%)	13 (18.1%)	

Data are mean ± SD, number (%), or median (interquartile range)

*ASD* absolute standardized difference (an ASD of ≥0.327 is considered imbalanced between the two groups), *eGFR* estimated glomerular filtration rate, *SBP* systolic blood pressure, *MAP* mean arterial pressure, *HR* heart rate, *PADUA* Preoperative Aspects and Dimensions Used for an Anatomical scores

^a^Included asthma (2 patients), chronic obstructive pulmonary disease (1 patient), obstructive sleep apnea (3 patients), history with lobectomy (2 patients), metastatic lung cancer (1 patient)

^b^Included coronary atherosclerotic heart disease (3 patients), gout (3 patients), lower limb artery thrombosis (1 patient), vasovagal syncope (1 patient), and liver disease (3 patients)

^c^Calculated according to the Chronic Kidney Disease Epidemiology Collaboration (CKD-EPI) equation [22]

^d^Measured in the operating room before anesthesia induction

^e^PADUA scores, a simple anatomical system used to predict the risk of perioperative surgical and medical complications in patients undergoing nephron-sparing surgery. The score ranges from 6 to 14, a score ≥ 8 indicates higher risk of complications [20]

^f^According to pathological examination after surgery

^g^Included chromophobe cell carcinoma (2 patients), papillary carcinoma (4 patients), malignant epithelial tumor (2 patients), cystic lesions (4 patients), oncocytic carcinoma (3 patients), unclassified renal cell carcinoma (5 patients), and inflammatory lesion (1 patient)

As expected, patients in the intervention group were given more dobutamine (*P* <  0.001) and norepinephrine (*P* = 0.028) during surgery when compared with the control group; they received more intraoperative fluid infusion (*P* = 0.012) and gave more urine output (*P* = 0.020). After reperfusion, SBP and MAP were higher, and heart rate (HR) was faster in the intervention group than in the control group (all *P* < 0.001). Other intraoperative variables were comparable between the two groups. For patients in the intervention group, the mean CI was higher whereas the mean SVV was lower after reperfusion than that before clamping (both P < 0.001) (Table 2).

**Table 2:** Intra−/postoperative variables

	Intervention group (***n*** = 72)	Control group (***n*** = 72)	***P*** value
Airway management			> 0.999
Endotracheal tube	59 (81.9%)	59 (81.9%)	
Laryngeal mask	13 (18.1%)	13 (18.1%)	
Intraoperative anesthetics			
Use of midazolam	18 (25%)	21 (29.2%)	0.574
Use of etomidate	30 (41.7%)	35 (48.6%)	0.402
Use of cisatracurium	50 (69.4%)	47 (65.3%)	0.594
Propofol (mg)	537 (393, 686)	510 (365, 650)	0.562
Sufentanil (μg)	30 (20, 38)	25 (20, 39)	0.689
Remifentanil (μg)	560 (0, 837)	562 (30, 815)	0.781
Rocuronium (mg)	50 (40, 50)	50 (40, 50)	0.998
Vasoactive drugs			
Use of dobutamine	65 (90.3%)	0 (0.0%)	**< 0.001**
Use of norepinephrine	6 (8.3%)	0 (0.0%)	**0.028**
Use of ephedrine	28 (38.9%)	23 (31.9%)	0.384
Use of diuretics during surgery	54 (75.0%)	56 (77.8%)	0.695
Mannitol (g)	25 (25, 25) (*n* = 39)	25 (25, 25) (*n* = 37)	0.201
Furosemide (mg)	10 (10, 10) (*n* = 49)	10 (10, 10) (*n* = 52)	0.215
Dexamethasone (mg)	5 (5, 5)	5 (5, 5)	0.229
Fluids infusion (ml)^a^	1650 (1525, 2100)	1600 (1213, 1800)	**0.012**
Urine output (ml)	400 (200, 600)	300 (200, 488)	**0.020**
Estimated blood loss (ml)	50 (30, 100)	50 (23, 100)	0.885
Packed red blood cells	1 (1.4%)	1 (1.4%)	> 0.999
Duration of anesthesia (min)	131 ± 42	135 ± 49	0.649
Type of surgery			> 0.999
Laparoscopic	64 (88.9%)	64 (88.9%)	
Open-abdominal	8 (11.1%)	8 (11.1%)	
Surgical position			> 0.999
Supine	3 (4.2%)	3 (4.2%)	
Lateral	69 (95.8%)	69 (95.8%)	
Duration of clamping (min) (*n* = 71)^b^	22 ± 8	22 ± 6	0.848
Before clamping^b^			
Mean BIS	50 ± 7	51 ± 7	0.348
Mean CI (L/min/m^2^)	2.7 (2.3, 3.1)	–	–
Mean SVV (%)	6 (5, 8)	–	–
Mean SBP (mmHg)	121 ± 15	120 ± 16	0.833
Mean MAP (mmHg)	88 ± 10	86 ± 9	0.181
Mean HR (beats per minute)	62 ± 8	60 ± 9	0.362
After reperfusion^b^			
Mean BIS	53 ± 8	51 ± 7	0.134
Mean CI (L/min/m^2^)	3.3 (3, 3.5) ‡	–	–
Mean SVV (%)	5 (4, 6) ‡	–	–
Mean SBP (mmHg)	136 ± 9	120 ± 15	**< 0.001**
Mean MAP (mmHg)	100 ± 4	86 ± 10	**< 0.001**
Mean HR (beats per minute)	70 ± 10	63 ± 8	**< 0.001**
Duration of surgery (min)	93 ± 38	96 ± 45	0.629
Use of diuretics within 3 day after surgery^c^	2 (2.8%)	1 (1.4%)	0.560

Data are mean ± SD, number (%), or median (interquartile range)

*SBP* systolic blood pressure, *MAP* mean arterial pressure, *HR* heart rate, *BIS* bispectral index, *CI* cardiac index, *SVV* stroke volume variation

^a^Only crystalloid fluid was allowed except blood transfusion

^b^One patient in the intervention group did not undergo renal artery clamping; one in the control group converted to radical nephrectomy without renal artery clamping

‡ *P* < 0.001 compared with value before clamping within the intervention group (analyzed with Wilcoxon signed-rank test)

^c^Including torasemide and furosemide

AKI developed in 12.5% (9/72) of patients in the intervention group and in 20.8% (15/72) of patients in the control group; the relative reduction of AKI was 39.9% in the intervention group but difference was not statistically significant (relative risk [RR] 0.60, 95% CI 0.28–1.28; *P* = 0.180). Per-protocol analysis also showed no significant difference between groups (12.7% [9/71] vs. 21.1% [15/71], RR 0.60, 95% CI 0.28–1.28; *P* = 0.179) (Table 3).

**Table 3:** Efficacy outcomes

	Intervention group (***n*** = 72)	Control group (***n*** = 72)	Relative risk or median difference (95% CI)^**a**^	***P*** value
**Primary endpoint**				
Acute kidney injury (ITT analysis)	9 (12.5%)	15 (20.8%)	RR = 0.60 (0.28–1.28)	0.180
Acute kidney injury (PP analysis)	9 (12.7%) (n = 71)	15 (21.1%) (n = 71)	RR = 0.60 (0.28–1.28)	0.179
**Secondary endpoints**				
Acute kidney injury classification				0.283
None	63 (87.5%)	57 (79.2%)		
Stage 1	9 (12.5%)	12 (16.7%)		
Stage 2	0 (0.0%)	2 (2.8%)		
Stage 3	0 (0.0%)	1 (1.3%)		
Complications within 30 days	4 (5.6%)	5 (6.9%)	RR = 0.80 (0.22–2.86)	> 0.999
Postoperative bleeding^b^	2 (2.8%)	1 (1.4%)		
Acute coronary syndrome^c^	1 (1.4%)	0 (0.0%)		
Surgical infection^d^	1 (1.4%)	4 (5.6%)		
Length of hospital stay after surgery (day)	4 (4, 5)	4 (4, 5)	MD = 0.0 (0.0–0.0)	0.541
30-day mortality	0 (0.0%)	0 (0.0%)	–	> 0.999
6-month mortality	0 (0.0%)	1 (1.4%)	–	> 0.999

Data are number (%), mean ± SD, or median (interquartile range)

*ITT* intention-to treat, *PP* per-protocol

^a^Calculated as the intervention group vs. or minus the control group

^b^Continued decrease of hemoglobin level and required blood transfusion and/or transarterial embolization

^c^Non-ST elevation myocardial infarction, diagnosed according to serum cardiac troponin I elevation and echocardiographic examination

^d^Fever > 38 °C, increased white blood cell count (> 12 × 10^9^/L) and elevated inflammatory biomarkers necessitating upgrading antibiotic treatment

**Fig. 2:**
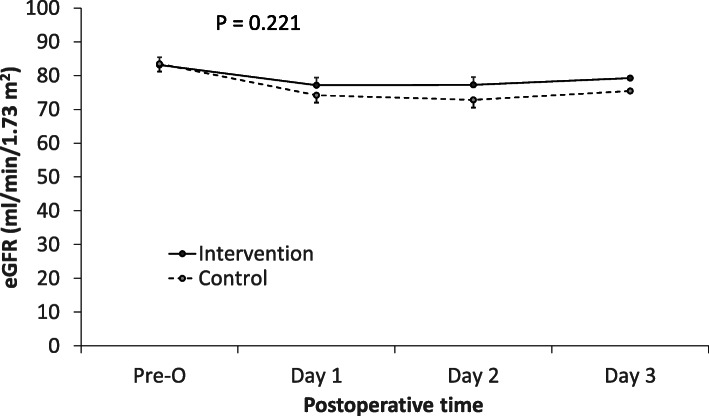
eGFR changes over time between groups. *P* = 0.221 (two-factor repeated measures ANOVA). eGFR, estimated glomerular filtration; calculated according to the Chronic Kidney Disease Epidemiology Collaboration (CKD-EPI) equation [22]

Regarding secondary endpoints, there were no significant differences in AKI classification, incidence of postoperative 30-day complications, postoperative length of hospital stay, as well as 30-day and 6-month mortality between groups (Table 3). None of patient who developed AKI received renal replacement therapy during the postoperative follow-up period. No significant difference was seen in eGFR change over time between two groups (Fig. 2). Safety outcomes from anesthesia induction to 2 h after surgery did not differ between two groups (Table 4).

**Table 4:** Safety outcomes^a^

	Intervention group (***n*** = 72)	Control group (***n*** = 72)	***P*** value
Hypertension^b^	19 (26.4%)	11 (15.3%)	0.101
Hypotension^c^	11 (15.3%)	19 (26.4%)	0.101
Tachycardia^d^	7 (9.7%)	4 (5.6%)	0.347
Bradycardia^e^	26 (36.1%)	30 (41.7%)	0.494
Frequent ventricular premature beat^f^	0 (0.0%)	1 (1.4%)	> 0.999
Massive bleeding^g^	0 (0.0%)	2 (2.8%)	0.497
Airway spasm^h^	2 (2.8%)	1 (1.4%)	> 0.999
Respiratory alkalosis^i^	0 (0.0%)	1 (1.4%)	> 0.999

Data are presented as number of patients (percentage)

^a^Safety outcome were monitored from anesthesia induction to 2 h after surgery

^b^Systolic blood pressure > 180 mmHg or an increase of more than 30% from baseline

^c^Systolic blood pressure < 90 mmHg or a decrease of more than 30% from baseline

^d^Heart rate > 100 bpm or an increase of more than 30% from baseline

^e^Heart rate < 50 bpm or a decrease of more than 30% from baseline

^f^New-onset ventricular premature beat that required antiarrhythmic therapy

^g^Volume of intraoperative bleeding > 1000 ml

^h^Wheezing rales heard in the lung field, with or without decrease of SpO_2_; relieved after intravenous corticosteroids or aminophylline

^i^Diagnosed according to blood gas results, occurred within 1 h after surgery in the ward and relieved after mask oxygen inhalation

## Discussion

Results of this pilot trial showed that, for patients undergoing partial nephrectomy for renal cancer, goal-directed fluid and blood pressure management reduced AKI by about 40%. However, the trial was under-powered. Large randomized controlled trials are required to confirm our results.

In the present study, AKI occurred in 20.8% of control group patients. This was lower than we expected [5], but was still within the reported range [5–8, 24, 25]. Two reasons might explain the unexpected lower incidence of AKI in our control group patients. The first one is the diagnostic criteria. AKI is usually diagnosed within 7 postoperative days according to the KDIGO criteria. In the present study, the majority of our patients were discharged within 3 to 4 days after partial nephrectomy. Furthermore, diuretics were commonly used during the perioperative period, these made urine output an unreliable parameter for AKI diagnosis; and early urine catheter removal made it difficult to monitor urine output per hour in the ward. Therefore, we diagnosed AKI only according to serum creatinine change within 3 postoperative days. This might have underestimated the rate of AKI development. However, recent studies also showed that the majority of surgery-related acute kidney injury occurred within 48 h of surgery [26]. Secondly, the improvement of surgeons’ skill and surgical technique helped preserve renal function. For example, the durations of renal hilus clamping and surgery were shorter in our patients than in previous studies [8, 24, 25].

Routine circulatory management during partial nephrectomy is to maintain blood pressure change within 20% from baseline and urine output > 0.5 ml/kg/h. However, the incidence of AKI remains high after surgery [8, 24, 25]. Experience from kidney transplantation suggested that maintaining adequate renal hydration and higher blood pressure after reperfusion (i.e., CVP > 8 mmHg and MAP > 95 mmHg) are beneficial for graft function [14, 15, 18]. Similar hemodynamic therapy may also relieve ischemia-reperfusion injury and protect renal function after partial nephrectomy.

Kidney is more sensitive to inadequate hydration compared with other organs. As Myles et al. [27] reported, restrictive fluid therapy is associated with a higher risk of AKI in renal transplant recipients. Static cardiac filling pressures such as CVP correlate poorly with the intravascular volume [28]; and hydration according to static parameters may induce excessive fluid infusion [29]. Better hemodynamic monitoring can be achieved with LiDCO^rapid^, a minimal invasive device that can monitor SVV, cardiac output and cardiac index through pressure contour analysis [30]. As a dynamic parameter, SVV is capable to reflect volume responsiveness and replace CVP [19, 28]. It was found that the optimal cutoff value of SVV is 6% and can be used as an alternative to CVP of 8 mmHg during kidney transplantation [19]. Therefore, SVV was maintained < 6% in this pilot trial as a hydration goal.

Cardiac output is an indicator of oxygen delivery and organ perfusion but is often compromised during general anesthesia. Studies showed that low-dose inotropic therapy is associated with an improved global oxygen delivery and tissue oxygenation [11]. However, in the study of Pearse et al. [12], cardiac-output guided hemodynamic management did not reduce complications including AKI after major gastrointestinal surgery. To be noted, dopexamine, a β2-agonist with both inotropic and vasodilator effects, was infused to obtain cardiac inotropy in the above study; blood pressure was ignored and might even be lower than usual due to the vasodilator effects of dopexamine, and as a result renal perfusion pressure was not guaranteed. In clinical practice, dopamine is also frequently used to increase blood pressure during kidney transplantation. But studies indicate that dopamine does not improve kidney function; on the contrary, it may produce potential harmful effects [31]. In this pilot study, dobutamine was adopted to maintain normal cardiac output and MAP > 95 mmHg in the intervention group; norepinephrine was infused if necessary.

This pilot study was the first to explore the effect of goal-directed circulatory management on renal function after partial nephrectomy. It seems that circulatory management with the goals of SVV < 6%, MAP > 95 mmHg and CI 3.0–4.0 L/min/m^2^ based on LiDCO^rapid^ hemodynamic monitoring didn’t significantly reduce postoperative AKI when compared with routine circulatory management, very possibly due to under-powered sample size. However, the relative risk reduction of AKI approaches 40%, which cannot be ignored and is clinically important. Our trial was underpowered because AKI incidence was lower than expected, and intervention reduced AKI by 40% rather than anticipated 52%. With the baseline AKI incidence of 20.8% and treatment effect of 40%, 626 patients would be required to provide 80% power. Further studies with larger sample sizes are needed to confirm our results.

Our study confirmed that patients’ overall renal function declined after surgery and, of those who developed AKI, most had mild renal injury. Our results were similar to previous studies [8]. Severe AKI is associated with increased mortality [32]; furthermore, mild AKI also negatively affected long-term functional recovery after partial nephrectomy and may increase the proportion of CKD upstaging [33, 34].

There are some limitations in this trial. Firstly, as a single-center study, the generalizability of our results may be limited. Secondly, interventions could not be blinded to anesthesiologists taking care of patients, which may bring bias. To reduce the related bias, anesthesiologists did not participate in patient recruitment and postoperative follow-up; whereas investigators who performed follow-ups were masked from study group assignment. Thirdly, AKI was diagnosed only according to serum creatinine level. This might have underestimated the real rate of AKI. Lastly, as a pilot study, the limited sample size diminished study power.

## Conclusions

For patients undergoing partial nephrectomy, goal-directed circulatory management to maintain SVV < 6%, MAP > 95 mmHg and CI 3.0–4.0 L/min/m^2^ from renal artery clamping to the end of surgery reduced postoperative AKI by 40%, although not significantly so. Further studies with larger sample sizes are required.

## Supplementary Information

**Additional file 1.**

## Data Availability

The datasets used and/or analyzed during the current study are available from the corresponding author on reasonable request.

## Abbreviations

AKI: Acute kidney injury
IRI: Ischemia-reperfusion injury
CVP: Central venous pressure
MAP: Mean arterial pressure
eGFR: estimated glomerular filtration rate
PADUA score: Preoperative aspects and dimensions used for an anatomical score
SVV: Stroke volume variation
CI: Cardiac index
KDIGO: the Kidney Disease: Improving Global Outcomes
CKD-EPI: the Chronic Kidney Disease Epidemiology Collaboration
HR: Heart rate
ASD: Absolute standardized difference
SBP: Systolic blood pressure
ITT: Intention-to-treat
PP: Per-protocol
