# Supplementary material for: Impact of goal-directed hemodynamic management on the incidence of acute kidney injury in patients undergoing partial nephrectomy: a pilot randomized controlled trial
Source: BMC Anesthesiol. 2021 Mar 3;21:67. doi: 10.1186/s12871-021-01288-8 (PMC7927248; doi:10.1186/s12871-021-01288-8)
Supplement: Supplementary file 1 — Additional file 1. [file 12871_2021_1288_MOESM1_ESM.docx]

**Telephone Interview at 30 days**

Patient ID：__________ Investigator：__________ Date：__________ (yyyy/mm/dd)

1. Is the patient alive？

□ Yes

□ No: Date of death：__________ (yyyy/mm/dd)

Reason：___________________________________

2. Did the patient re-examine serum creatinine?

□ No

□ Yes

Date of re-examination：_________ (yyyy/mm/dd)

Medical institution：_________ Result：__________

1. Did the patient need renal replacement?

□ No

□ Yes

1. Did the patient develop any complications?
2. Acute myocardial infarction/angina pectoris

□ No

□ Yes (Hospital re-admission: □ No; □ Yes: date: ___________________________)

1. Respiratory infection

□ No

□ Yes (Hospital re-admission: □ No; □ Yes: date: ___________________________)

1. Stroke

□ No

□ Yes (Hospital re-admission: □ No; □ Yes: date: ___________________________)

1. Infection in the operating site

□ No

□ Yes (Hospital re-admission: □ No; □ Yes: date: ___________________________)

1. Surgical bleeding

□ No

□ Yes (Hospital re-admission: □ No; □ Yes: date: ___________________________)

1. Did the patient complaint any other discomfort?

□ No

□ Yes (specify: _________________________________________________________)

1. Was the patient re-admitted to hospital due to other reasons (including surgery)?

□ No

□ Yes (specify: _________________________________________________________)

**Telephone Interview at 3 months**

Patient ID：__________ Investigator：__________ Date：__________ (yyyy/mm/dd)

1. Is the patient alive？

□ Yes

□ No: Date of death：__________ (yyyy/mm/dd)

Reason：___________________________________

2. Did the patient re-examine serum creatinine?

□ No

□ Yes

Date of re-examination：_________ (yyyy/mm/dd)

Medical institution：_________ Result：__________

1. Did the patient need renal replacement?

□ No

□ Yes

**Telephone Interview at 6 months**

Patient ID：__________ Investigator：__________ Date：__________ (yyyy/mm/dd)

1. Is the patient alive？

□ Yes

□ No: Date of death：__________ (yyyy/mm/dd)

Reason：___________________________________

2. Did the patient re-examine serum creatinine?

□ No

□ Yes

Date of re-examination：_________ (yyyy/mm/dd)

Medical institution：_________ Result：__________

1. Did the patient need renal replacement?

□ No

□ Yes
